# Cervical Approach for Ectopic Mediastinal Goiter: A Case Report

**DOI:** 10.7759/cureus.82680

**Published:** 2025-04-21

**Authors:** Sara Zarrouki, Doaae El Ouaddane, Rachid Marouf

**Affiliations:** 1 Thoracic Surgery, Faculty of Medicine and Pharmacy, Mohammed VI University Hospital, Mohammed First University, Oujda, MAR

**Keywords:** cervical approach, ectopic goiter, mediastinal blood supply, mediastinal mass, primary mediastinal goiter

## Abstract

Ectopic mediastinal goiter (EMG) is an unusual condition where thyroid tissue develops in the mediastinum instead of its usual location in the neck. Diagnosing EMG can be challenging, as it represents only a small fraction of all mediastinal tumors. Management typically involves a sternotomy or other thoracic approaches; however, a cervical approach may be considered in select cases. We report the case of a 65-year-old woman who was admitted following the incidental discovery of an anterior mediastinal mass on chest CT. The mass displayed imaging characteristics similar to thyroid tissue but appeared independent of the cervical thyroid gland. Surgical removal was successfully performed via a cervical approach, and histopathological analysis confirmed the diagnosis of an ectopic colloid goiter. EMG is distinct from secondary retrosternal goiters due to the lack of continuity with the cervical thyroid. It is often asymptomatic but may present with compressive symptoms depending on its size and location. Imaging studies play a key role in diagnosis, although differentiating EMG from other mediastinal masses can be difficult. Surgical excision is generally required to prevent compressive complications. While sternotomy and thoracic approaches remain standard, our case - and others in the literature - demonstrates that a cervical approach with meticulous dissection may be sufficient, particularly for masses located in the anterior or superior mediastinum. EMG should be considered in the differential diagnosis of mediastinal masses, and a cervical approach offers a less invasive alternative for appropriately selected patients.

## Introduction

Ectopic mediastinal goiter (EMG) is a rare condition characterized by the presence of thyroid tissue within the mediastinum, often presenting as a mediastinal mass. Ectopic thyroid tissue results from aberrant embryogenesis and may be located anywhere along the thyroid’s migratory path, from the tongue to the diaphragm [1]. The most common site is the lingual thyroid, which accounts for approximately 90% of ectopic thyroid cases, with a reported prevalence of 1 in 100,000-300,000 individuals [1]. In contrast, EMG is much less common, representing only about 1% of all mediastinal tumors [2,3].

Given its rarity and variable clinical presentation, EMG can be difficult to diagnose and is often overlooked in the differential diagnosis of mediastinal masses. Accurate identification is essential, as the choice of treatment can have a significant impact on patient outcomes.

Surgical management of EMG typically involves sternotomy or other thoracic approaches. However, in selected cases, alternative techniques may be feasible. Here, we report a case of EMG that was successfully excised through a cervical approach.

## Case presentation

A 65-year-old woman with a medical history of diabetes, hypertension, and glaucoma was admitted following the incidental discovery of an anterior mediastinal mass on a chest CT scan. Physical examination revealed bilateral nodules on cervical palpation; however, there were no clinical signs of dysthyroidism. Thyroid function tests showed subclinical hyperthyroidism, with a low thyroid-stimulating hormone level of 0.03 mU/mL (reference range: 0.27-4.2) and a normal free thyroxine level of 15.06 pmol/L (reference range: 12-22; Table 1). Antibody tests for anti-thyroid peroxidase and anti-thyroglobulin were negative, and other laboratory findings were within normal limits.

**Table 1:** Thyroid function test results FT4, free thyroxine; TSH, thyroid-stimulating hormone

Blood test	Patient value	Reference range
TSH (mU/mL)	0.03	0.27-4.2
FT4 (pmol/L)	15.06	12-22

A cervical ultrasound revealed an enlarged thyroid gland with multiple bilateral nodules, classified as TIRADS 3. The largest nodule was located at the inferior pole of the right lobe, measuring 36.8 × 20 × 38.5 mm. Another nodule was identified at the superior pole of the left lobe, measuring 12.7 × 12 × 22.7 mm. In addition to these findings, the ultrasound detected a distinct, heterogeneous mass in the superior mediastinum, displaying an echotexture similar to that of thyroid tissue.

**Figure 1:**
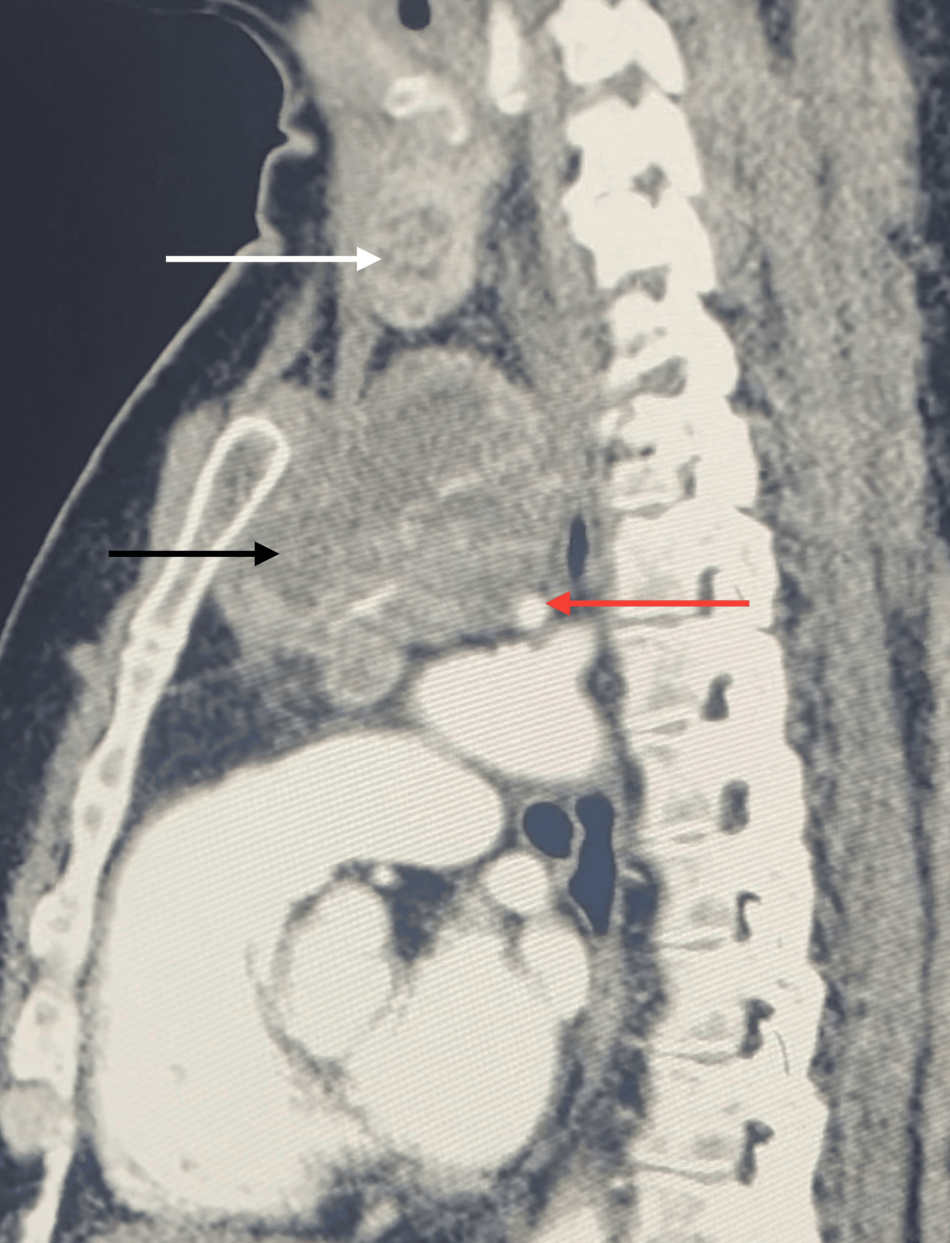
CT scan showing the EMG (black arrow) with its mediastinal blood supply (red arrow), separated from the thyroid gland (white arrow) EMG, ectopic mediastinal goiter

Further imaging with a CT scan confirmed the presence of a large anterior mediastinal mass, measuring 61 × 48 × 69 mm, separate from the cervical thyroid gland. The mass shared imaging characteristics with thyroid tissue and was compressing the trachea, as well as the left jugular vein and the innominate trunk (Figure 1).

**Figure 2:**
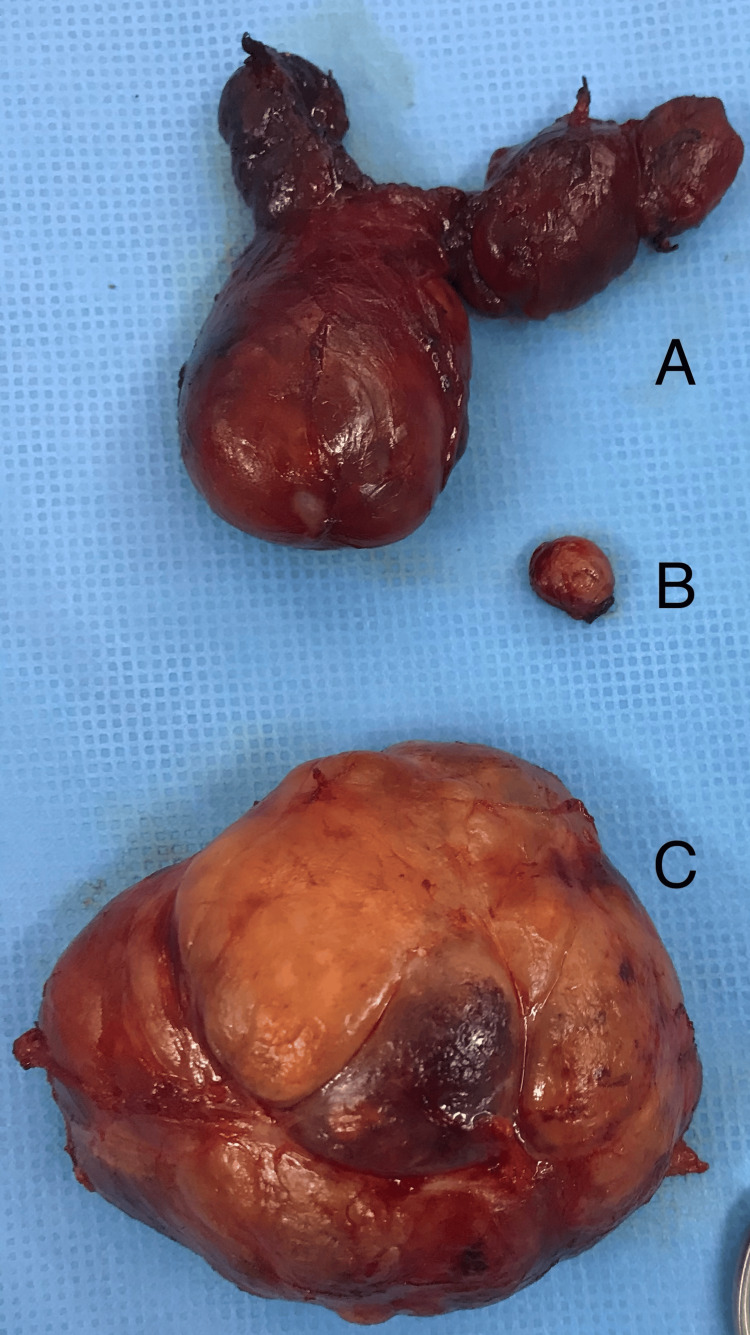
Surgical specimen: (A) cervical thyroid gland, (B) left cervical thyroid nodule, and (C) mediastinal mass (EMG) EMG, ectopic mediastinal goiter

Surgical removal was planned due to the compressive nature of the mediastinal mass observed on the CT scan. The first stage of the procedure involved a thyroidectomy performed via a cervical approach. Following thyroidectomy, the mediastinal mass - separate from the cervical thyroid - became accessible. This approach allowed for complete resection of the mass without the need for partial or total sternotomy (Figure 2).

Postoperatively, the mediastinal drain was removed two days after surgery, and the patient was discharged 24 hours later. Histological examination of the thyroid and the left nodule revealed nodules surrounded by a thin capsule, composed of vesicles of varying sizes with regular epithelial lining and abundant colloid. The mediastinal mass exhibited similar histological features, confirming the diagnosis of ectopic goiter.

The patient was started on L-thyroxine, with the dosage adjusted according to follow-up thyroid hormone levels. Her postoperative course was uneventful, with no clinical or radiological abnormalities observed during follow-up.

## Discussion

EMG is a rare entity; more than 95% of mediastinal goiters are secondary to the downward extension of the cervical thyroid gland, while true EMG accounts for only 1.7% of cases [4].

The distinction between ectopic mediastinal thyroid tissue - also referred to as primary mediastinal goiter - and secondary retrosternal goiter is based on two main criteria: the source of blood supply and the presence or absence of a connection between the cervical thyroid gland and the mediastinal mass. Secondary retrosternal goiter arises from the downward growth of the thyroid gland into the mediastinum. In contrast, primary mediastinal goiter, which occurs in fewer than 1% of cases, originates from ectopic thyroid tissue with no anatomical connection to the cervical thyroid gland [4-6].

This ectopic thyroid tissue is typically vascularized by mediastinal vessels, such as the aorta, internal mammary arteries, or one of the supra-aortic trunks [7]. Mediastinal ectopic goiter (MEG) is often asymptomatic and is frequently discovered incidentally during CT scans performed for unrelated reasons. However, depending on its size and location, MEG can compress adjacent mediastinal structures, leading to symptoms such as dysphagia, respiratory difficulty, cough, chest pain, or vascular compression [8].

Many asymptomatic cases are initially suspected due to a widened superior mediastinum observed on chest X-ray [9]. The imaging appearance of EMG can be misleading and may be confused with more common mediastinal masses such as lymphoma, thymoma, or teratoma. Nonetheless, CT imaging plays a crucial role in differentiating ectopic thyroid tissue from other mediastinal tumors, particularly due to its vascular characteristics, as demonstrated in our patient [10]. In most cases of retrosternal goiter, especially in women and patients over 50 years old, the diagnosis is clinical and does not typically require histological confirmation prior to surgery [11].

On MRI, ectopic thyroid tissue usually appears as a round mass with high signal intensity on both T1- and T2-weighted images [12]. Surgical treatment is strongly recommended to prevent compressive complications, address potential malignancy, and when biopsy is inconclusive or not feasible [11]. In cases of bilateral goiter, thyroidectomy is generally advised [11].

To date, five cases of EMG have been successfully excised via a cervical approach [7-9]. These cases shared a common feature: the location of the mass in the anterior and/or superior mediastinum, which made it accessible through a cervical incision.

Several authors advocate for an additional incision during the cervical approach when managing primary mediastinal goiter to ensure complete resection and minimize the risk of bleeding complications [13,14]. Among these authors, sternotomy is often favored as it provides better access and control over the mediastinal blood supply.

However, based on our case and previously reported experiences (Table 2), we propose that a cervical approach, combined with careful blunt dissection, can be sufficient - provided that the ectopic mediastinal thyroid is accessible and its vascular supply can be adequately managed [8]. Nonetheless, it is advisable to plan for the possibility of an additional incision, such as a sternotomy or thoracic approach, to address any intraoperative complications or unexpected findings.

**Table 2:** Reported cases of EMG removed via cervical approach EMG, ectopic mediastinal goiter

Reference	Series size	Mediastinal location	Vascular supply	Histology	Previous thyroid surgery
Walz et al. (2013) [7]	1	Anterior	Mediastinal	Adenomatous nodular hyperplasia	No
Nakaya et al. (2017) [8]	2	Anterior, middle	Not specified	Adenomatous goiter (two cases)	NS
Motlaghzadeh et al. (2023) [9]	2	Superior (two cases)	Not specified	Not specified	Not specified

## Conclusions

EMG is a rare but important differential diagnosis for mediastinal masses. While it can often be identified through clinical assessment and imaging, it is sometimes overlooked, which may lead to delays in diagnosis. Traditionally, thoracic approaches - such as sternotomy or thoracotomy - have been considered the safest and most reliable by many surgeons. However, in carefully selected cases, a cervical approach may be sufficient, depending on the extent of mediastinal involvement and the accessibility of the mass’s vascular supply.
